# Nutritional perspectives on sickle cell disease in Africa: a systematic review

**DOI:** 10.1186/s40795-021-00410-w

**Published:** 2021-03-18

**Authors:** Eunice Berko Nartey, Jonathan Spector, Seth Adu-Afarwuah, Catherine L. Jones, Alan Jackson, Agartha Ohemeng, Rajiv Shah, Alice Koryo-Dabrah, Amma Benneh-Akwasi Kuma, Hyacinth I. Hyacinth, Matilda Steiner-Asiedu

**Affiliations:** 1 Department of Nutrition and Food Science, University of Ghana, Legon, Ghana; 2 Department of Nutrition and Dietetics, University of Health and Allied Sciences, PMB 31, Ho, V/R Ghana; 3 Novartis Institute for Tropical Diseases, Cambridge, USA; 4 Novartis Institute for Tropical Diseases, Emeryville, CA USA; 5 Emeritus Professor of Human Nutrition, Southampton General Hospital (MP 113), Tremona Road, Southampton, SO16 6YD UK; 6 Novartis Global Health and Corporate Responsibility, Forum 1, Fabrikstrasse, Basel, Switzerland; 7 Department of Hematology, School of Medicine and Surgery, University of Ghana, Korle-Bu, Ghana; 8 Aflac Cancer and Blood Disorder Center of Children’s Healthcare of Atlanta and Emory University Department of Pediatrics, Atlanta, GA USA; 9 The Atlanta Sickle Cell Disease Consortium, Atlanta, USA

**Keywords:** Nutritional status, Malnutrition, Sickle cell disease, Sickle cell anemia, Systematic review

## Abstract

**Background:**

Sickle cell disease (SCD) is an inherited blood disorder that predominantly affects individuals in sub-Saharan Africa. However, research that elucidates links between SCD pathophysiology and nutritional status in African patients is lacking. This systematic review aimed to assess the landscape of studies in sub-Saharan Africa that focused on nutritional aspects of SCD, and highlights gaps in knowledge that could inform priority-setting for future research.

**Methods:**

The study was conducted using the Preferred Reporting Items for Systematic Reviews and Meta-Analysis (PRISMA) guidelines. Inclusion criteria comprised original, peer-reviewed research published between January 1995 and November 2020 involving individuals in Africa with any phenotypic variant of SCD and at least one nutritional status outcome. Nutritional status outcomes were defined as those that assessed dietary intakes, growth/anthropometry, or nutritional biomarkers. Databases used were Ovid Embase, Medline, Biosis and Web of Science.

**Results:**

The search returned 526 articles, of which 76 were included in the final analyses. Most investigations (67%) were conducted in Nigeria. Studies were categorized into one of three main categories: descriptive studies of anthropometric characteristics (49%), descriptive studies of macro- or micronutrient status (41%), and interventional studies (11%). Findings consistently included growth impairment, especially among children and adolescents from sub-Saharan Africa. Studies assessing macro- and micronutrients generally had small sample sizes and were exploratory in nature. Only four randomized trials were identified, which measured the impact of lime juice, long-chain fatty acids supplementation, ready-to-use supplementary food (RUSF), and oral arginine on health outcomes.

**Conclusions:**

The findings reveal a moderate number of descriptive studies, most with small sample sizes, that focused on various aspects of nutrition and SCD in African patients. There was a stark dearth of interventional studies that could be used to inform evidence-based changes in clinical practice. Findings from the investigations were generally consistent with data from other regional settings, describing a significant risk of growth faltering and malnutrition among individuals with SCD. There is an unmet need for clinical research to better understand the potential benefits of nutrition-related interventions for patients with SCD in sub-Saharan Africa to promote optimal growth and improve health outcomes.

## Background

Sickle cell disease (SCD) is the most common inherited blood disease worldwide, with the vast majority of cases occurring in sub-Saharan Africa [1]. The condition derives from a point mutation of the β-globin gene found on the short arm of chromosome 11 through which the hydrophilic amino acid glutamic acid is substituted with the hydrophobic amino acid valine at the sixth position [2, 3]. The result is a change in the structure and dynamics of hemoglobin such that certain conditions including deoxygenation and acidosis predispose to hemoglobin polymerization. When this occurs, erythrocytes assume a misshapen and rigid form that promotes pathological processes leading to intravascular inflammation and occlusion of small blood vessels [4]. Since these processes can take place anywhere in the body, the disease is highly complex and characterized by multiple potential life-threatening complications that include acute splenic sequestration, aplastic crises, acute chest syndrome, infection, heart failure, and stroke [5, 6]. The disease’s clinical hallmarks include acute painful crises and severe anemia [1]. In sub-Saharan Africa, it has been estimated that up to 90% of children born with SCD die before 5 years of age [7].

In high resource countries, mortality from SCD has decreased dramatically over the past five decades. The improvements in outcomes have been attributed in part to newborn screening and comprehensive care programs designed to prevent disease complications to the extent possible and to treat complications of disease when they occur [8]. Early detection of disease enables clinicians and families to institute measures to proactively prevent complications and facilitate timely treatment when needed. For example, the risk of fatal infection has been shown to be reduced through vaccination and administration of prophylactic antibiotics [9]. Furthermore, treatments with blood transfusion and hydroxyurea therapy has led to superior outcomes in the long term [10, 11]. Unfortunately, the availability of vaccines, medicines, and other interventions is highly variable in sub-Saharan Africa. Increasing access to proven preventative and treatment modalities is therefore an urgent priority [12]. At the same time, there is a need to identify new ways of maximizing the well-being of individuals with SCD in Africa and it is in this context that nutritional interventions could possibly play an important role.

There is evidence that the pathophysiology of SCD has substantial nutritional implications including higher energy and nutrients requirements, nutrient deficiencies, and growth abnormalities [13–15]. It is theorized that a main driver of disease complications is higher rates of metabolic expenditure in individuals with SCD resulting from increased hematopoiesis, increased cardiac output, chronic inflammation, and related processes [16, 17]. Since nutrition interventions could be a mechanism for addressing increased energy expenditure, attention to nutritional care is increasingly seen to be an important aspect of supportive management for patients with SCD [18, 19], especially in resource poor settings. However, evidence-based nutritional guidelines for patients with SCD in Africa are lacking and the extent of nutrition-focused research involving individuals in Africa with SCD is unclear. We undertook this systematic review to evaluate the existing literature focused on nutritional aspects of SCD in sub-Saharan Africa. Specifically, we sought to assess the number and nature of relevant studies, review their findings, and identify gaps in knowledge that could inform priority-setting for future research.

## Methods

### Eligibility criteria

We sought to include all studies involving original research that focused on the nutritional status of individuals with SCD in an African population. Studies involving nutritional status were defined as those that investigated topics of dietary intake, measurements of growth or anthropometry, or nutrition-related biomarkers. Studies that did not differentiate the cause of the anemia were excluded, as were studies that only included nutrition interventions as part of comprehensive care programmes (i.e., studies that did not clearly report nutrition-related outcomes). Studies involving both children and adults were included. The focus of this analysis was on studies involving individuals with various forms of SCD including HbSS, HbSC, and rarer genetic variants of disease; studies were excluded that only involved individuals with sickle cell trait. Case reports and review articles were also excluded.

### Informational sources and search strategy

The databases employed for this search were Ovid Embase, Medline, Biosis, and Web of Science. The date range was January 1st, 1995, through November 30th, 2020, such that the reference list covered a period of approximately 25 years. We performed a Boolean search using specific Boolean operators and the following search terms: “Sickle cell disease” or “sickle cell anemia” or “hemoglobinopathy” AND Africa or specific African countries (all African countries were individually listed) AND various nutrition-related terms (i.e., nutrition, growth, macronutrient, micronutrient, vitamin, mineral, anthropometric, height, length, weight, head circumference, mid-upper arm circumference, MUAC, dietary intake, recommended dietary allowance, RDA, nutritional status) along with associated terms (both indexed and non-indexed) for nutrition, diet and growth, and specific vitamins and minerals. We also allowed for inclusion of articles that were identified through review of the bibliographies of papers that underwent full-text review. Investigations written in any language were included provided sufficient translation into English could be assured.

### Data management and selection process

Titles and abstracts were each screened by two independent reviewers. Any title or abstract that appeared to meet inclusion criteria or for which there was uncertainty prompted a full text review. Reviewing of full text articles were assigned to individual investigators. If eligibility of a full text article was unclear, it was resolved by discussing it with at least two other reviewers on the research team who were not earlier assigned the full text article using the inclusion criteria. To maximize consistency among reviewers, each reviewer initially reviewed 10 articles and the review team together discussed the initial dataset that had been extracted to ensure accuracy and completeness. The review process then proceeded according to the process described.

### Data synthesis

Study data were extracted into standardized forms using Microsoft Excel (Microsoft, Redmond, USA) where they were organized for analyses. Depending on the nature of the articles that met inclusion criteria, we extracted information relating to geography, subject age group, sample size, comparison groups, biomarkers, and other relevant variables. Since the main purpose of this investigation was to broadly understand the types of nutrition-related studies that have been conducted involving individuals with SCD in Africa, we chose not to systematically judge the quality of evidence or risks of bias within individual studies. Rather, we discussed specific merits and limitations of individual studies where appropriate in the context of major themes that would emerge in the analyses. We planned for a quantitative categorization of the types of articles (e.g., descriptive versus interventional studies) and a narrative synthesis of data in table and text format to summarize and assess the results.

### PRISMA

The study was conducted and reported according to PRISMA (Preferred Reporting Items for Systematic reviews and Meta-Analyses) guidelines [20, 21].

## Results

### Search results

**Fig. 1:**
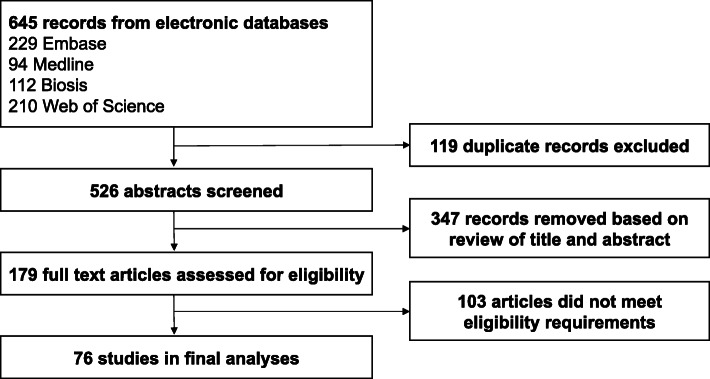
Literature search flowchart

In total, 526 unique titles and abstracts were identified through the literature search. Of those, 347 did not meet inclusion criteria and 179 full-text articles were assessed. Seventy-six studies were deemed eligible and included in the final analyses (Fig. 1).

### Results overview

Most investigations (67%) were conducted in a single country (Nigeria). Each study was placed into one of three main categories according to the primary nature of the investigation: (a) descriptive study of anthropometric characteristics (37 articles; 49%); (b) descriptive study of macro- or micronutrient status (31 articles; 41%); and (c) interventional studies (8 articles; 11%). The studies are summarized in Tables 1, 2, 3 and described in greater detail below.

**Table 1:** Studies of anthropometric characteristics of individuals living with sickle cell disease in Africa

Authors and year of publication	Location	Ages	No. of subjects	Control group	Weight	Height	Body mass index	Other assessments
VanderJagt et al., 1997 [22]	Jos, Nigeria	10 months-14 years (mean 7 years for males; mean 6 years for females)	13	17 age- and gender-matched controls	Significantly lower weight in males with SCD	No significant differences in height reported	No statistical difference in BMI as both weight and height were lower for SCD patients	• Significantly lower head circumference in males with SCD versus controls • No differences in MUAC or triceps skin fold • No differences reported for females
Soliman et al., 1999 [23]	Alexandria, Egypt	Mean 7 years	110	200 healthy age-matched children, 30 children with constitutional growth delay, 25 children with growth hormone deficiency	N/A	• Height and growth velocity significantly lower in SCD patients than healthy controls • Bone age delay in SCD patients by 2.4 years	• BMI not significantly different between SCD patients and healthy controls	• Lower MUAC and triceps skin fold thickness in SCD patients versus healthy controls • Significant age delays in puberty in SCD patients
Aderibigbe et al., 1999 [24]	Ilorin, Nigeria	18–54 years (mean 22 years)	64	60 adults aged 18–57	Significantly lower weight in individuals with SCD	Significantly shorter height in individuals with SCD	N/A	
VanderJagt et al., 2000 [25]	Jos, Nigeria	3–20 years	48	51 healthy controls	Significantly lower weight in males with SCD aged 10–18 years; no differences for females	No differences	Significantly lower BMI for males with SCD aged 10–18; no differences for females	• Significantly lower fat free mass in males with SCD aged 10–18 • Significantly higher body fat and % body fat in individuals with SCD aged > 10 years
Vandegt et al., 2002 [26]	Jos, Nigeria	Mean 13 years for males and females	72	68 age- and gender-matched controls	Significantly lower weight in females and males with SCD	Significantly lower height in females and males with SCD	Significantly lower BMI in females and males with SCD	
VanderJagt et al., 2002 [27]	Jos, Nigeria	8–22 years (mean 14 years for males; mean 13 years for females)	80	41 male and 38 female age-matched controls; 51 male and 71 female additional controls for ultrasound component	Significantly lower weight in females and males with SCD	Significantly lower height in females and males with SCD	Significantly lower BMI in males and females with SCD	• Significantly lower bone density in individuals with SCD by ultrasound measurement • Some differences in serum markers of bone resorption and formation • No differences in triceps skin fold • Significantly lower MUAC, FFM, and % FFM in males and females with SCD; males with SCD also had significantly lower body fat
Oredugba et al., 2002 [28]	Lagos, Nigeria	1–18 years (mean 10 years)	117	122 children and adolescents aged 1–18 years from well-baby clinics and surgical outpatient unit	Significantly lower weight in individuals with SCD aged 18 years	No differences	N/A	• Significantly lower MUAC in individuals with SCD • No differences in mean head circumference
Glew et al., 2003 [29]	Jos, Nigeria	10–18 years (mean 14 years for males; mean 13 years for females)	77	75 age- and gender-matched controls	Significantly lower weight in individuals with SCD	Significantly lower height in individuals with SCD	Significantly lower BMI in males with SCD; no differences for females	
VanderJagt et al., 2007 [30]	Jos, Nigeria	7–35 years (mean 15 years for males; mean 17 years for females)	102	104 healthy age and gender matched controls	Significantly lower weight in individuals with SCD	• Significantly lower mean sitting height in individuals with SCD • Significantly lower standing height in males with SCD (not significantly different for females)	Significantly lower BMI in females with SCD; no differences for males	• Significantly lower FFM in individuals with SCD; no differences in FFM% and fat % • Significantly lower phase angle (measure of overall nutritional status) in individuals with SCD • Significantly lower MUAC and triceps skin fold in females with SCD (not for males)
Aina et al., 2010 [31]	Lagos, Nigeria	10–19 years (mean 14 years)	136	136 age- and gender-matched controls	N/A	N/A	N/A	Delayed puberty in males and females with SCD
Cox et al., 2011 [32]	Dar es Salaam, Tanzania	6 months-48 years (mean 10 years)	1041	717 HbAA siblings, clinic walk-ins, and referrals	SCD status was significantly associated with underweight; adult males were more likely to be underweight than females	SCD was significantly associated with stunting; adult males were more likely to be stunted than females	SCD was significantly associated with wasting; adult males were more likely to have wasting than females	
Osei-Yeboah, 2011 [33]	Ghana	1–12 years (mean 7 years)	357; phenotype included SS, SC, SD, S Beta thal	70 HbAA siblings	• Significantly lower weight-for-age in individuals with SCD • Prevalence of malnutrition higher in individuals with SCD (61%) versus controls (29%) • No significant differences in rates of wasting (weight-for-height)	Prevalence of stunting higher in individuals with SCD (35%) versus controls (3%)	N/A	
Iwalokun et al., 2011 [34]	Lagos, Nigeria	5–35 years	55 (31 steady-state and 24 unstable/ crisis)	22 “non-SCD” individuals	Significantly lower weight for males > 16 years; not different in other age groups	N/A	Significantly lower BMI in females with SCD (all age groups) and males with SCD (> 16 years)	• Significantly lower fat mass in males with SCD (> 16 years); not significantly different for other age groups • Significantly lower leptin levels in males with SCD (≤16 years) and all female age groups
Animasahun et al., 2011 [35]	Lagos, Nigeria	1–10 years (mean 6 years)	100	100 individuals with phenotype HbAA matched by age, socio-economic class, and gender	Significantly lower mean weight and weight-for-height in individuals with SCD	Mean height showed no difference between SCD patients and controls	No difference in mean BMI	
Akodu et al., 2012 [36]	Lagos, Nigeria	2–15 years (mean 8 years)	80	80 individuals with phenotype HbAA	No statistical difference reported	N/A	Significantly lower BMI in individuals with SCD	
Tebbani et al., 2014 [37]	Annaba city, Algeria	6–12 years	30	WHO standard references	Lower weight in individuals with SCD compared with WHO standards	Height was below WHO standard references for SCD patients	N/A	
Akingbola et al., 2014 [38]	Ibadan, Oyo, Nigeria and Chicago, USA	11–30 years	214	209 individuals with SCD aged 11–30 years living in USA (compares characteristics of individuals with SCD in Nigeria to those in US)	Significantly lower weight in individuals with SCD in Nigeria vs US in patients ≥18 years old	Significantly lower height in individuals with SCD in Nigeria vs US in patients ≥18 years old	Significantly lower BMI in individuals with SCD in Nigeria vs US in patients ≥18 years old	
Akodu et al., 2014 [39]	Lagos, Nigeria	8 months-15 years (mean 6 years)	100	100 HbAA age- and sex-matched controls	N/A	Significantly lower sitting height in individuals with SCD aged > 10 years; height not significantly different	N/A	Significantly shorter arm span in individuals with SCD aged > 10 years
Tsang et al., 2014 [40]	Nyanza Province, Western Kenya	6–35 months	14	288 children from random sample of 882; Underweight, stunting and wasting were defined using WHO 2006 standards	No significant association of HbSS with underweight	No significant association of HbSS with stunting	No significant association of HbSS with wasting	
Eke et al., 2015 [41]	Enugu, Nigeria	6–18 years (mean 11 years)	132	132 age- and gender-matched HbAA children and adolescents from nearby schools	Significantly lower weight in females with SCD aged 10–18 years; no differences in males	No differences	Significantly lower BMI in females with SCD aged 10–18 years; no differences in males	• No differences in body fat % or visceral fat % • Significantly lower skeletal muscle % in males with SCD aged 6–9 years
Eke et al., 2015 [42]	Enugu, Nigeria	1–5 years (mean 3 years)	58	58 age- and gender-matched HbAA individuals	• Significantly lower weight-for-age in individuals with SCD • Significantly lower rate of obesity in individuals with SCD (3.4% vs 22.4%)	No difference in height-for-age	Significantly lower BMI in individuals with SCD	• Significantly lower weight-for-height in individuals with SCD
Ranque et al., 2016 [43]	Cameroon, Ivory Coast, Gabon, Mali, Senegal	10–24 years (median 16 years)	3627	943 controls aged 14–33 years; controls were significantly older (median age 24 years versus 16 years for SCD patients) and more likely to be female (60% vs 54%)	N/A	Significantly lower height in individuals with SCD	Significantly lower BMI in individuals with SCD	
Odetunde et al., 2016 [44]	Enugu State, Nigeria	6–20 years (mean 12 years)	40	40 age-, gender-, socioeconomic status-matched HbAA individuals from area schools	Significantly lower weight in individuals with SCD	No differences in height	48% with SCD were underweight (BMI < 5th percentile); 13% of controls were underweight	
Esezobor et al., 2016 [45]	Lagos, Nigeria	2–17 years (mean 9 years)	233	Compared with WHO 2007 standards	23% of individuals with SCD had wasting (low weight-for-height) or severe wasting	12% of SCD patients were stunted or severely stunted; 75.5% were normal height	2% of individuals with SCD were overweight or obese	
Senbanjo et al. 2016 [46]	Lagos, Nigeria	Children up to age 15 years (mean 7 years)	118 (114 HbSS and 4 HbSC phenotype)	118 age-, gender-, and socioeconomic class-matched; stunting/malnutrition based on WHO 1995 standards	Significantly higher rate of “thinning” in individuals with SCD aged 11–15 years	Significantly higher rate of stunting in individuals with SCD aged 11–15 years	N/A	No overall difference in mean head circumference
Oluwole et al., 2016 [47]	Lagos, Nigeria	6–16 years (mean 9 years)	56	44 individuals without SCD	Significantly lower weight-for-age in individuals with SCD	Significantly lower height in individuals with SCD	Significantly lower BMI in individuals with SCD	
Adegoke et al., 2017 [48]	Ilesa, Nigeria	4–11 years	95	109 Brazilian children with SCD aged 4–11 years; 36 were hydroxyurea (HU)-naive to match Nigerian patients (study compares SCD populations in Nigeria and Brazil)	30% of SCD patients in Nigeria had low weight-for-height; 4.3% were overweight or obese	13% of SCD patients in Nigeria were of short stature; 8.4% were tall for age; significantly lower mean height-for-age among Nigerian patients compared with Brazilian HU-naive patients	Significantly lower BMI in Nigerian individuals with SCD	Significantly lower triceps skin fold, upper arm area, upper arm muscle area, and fat % among Nigerian patients compared with the Brazilian HU-naive patients
Mikobi et al., 2017 [49]	Kinshasa, Democratic Republic of Congo	Mean 25 years	140	Study compared groups of SCD patients stratified by disease severity	N/A	N/A	Significantly lower BMI in patients with greater disease severity	
Kazadi et al., 2017 [50]	Kinshasa, Democratic Republic of Congo	Under 12 years	159	296 age-, gender-, and neighborhood-matched individuals with HbAA; comparisons focused on children under age 12 years; underweight, stunting and wasting were defined using WHO 2006 standards	Significantly lower weight in individuals with SCD (39.6% of individuals with SCD versus 12.2% of controls)	Significantly more stunting in individuals with SCD (34.6% in individuals with SCD versus 9.8% of controls)	N/A	Factors significantly associated (*P* < 0.01) with poor growth included frequency of crises, age < 1 yr. at first transfusion, and hand-foot syndrome
Sokunbi et al., 2017 [51]	Nigeria	5–18 years (mean 9 years)	175	175 age-matched HbAA individuals	No statistical difference reported	Significantly lower height in individuals with SCD	No statistical difference reported	
Onukwuli et al., 2018 [52]	Enugu, Nigeria	6–18 years (females only)	81 (females only)	81 age- and socioeconomic class-matched HbAA individuals recruited from outpatient clinic	Significantly lower mean weight in individuals with SCD	No differences in mean height	Significantly lower BMI in individuals with SCD	
Osei et al., 2019 [53]	Kumasi, Ghana	3–12 years	100; phenotypes included SS, Sβ^0^, SC, Sβ^+^	Compared with WHO growth standards	37% of individuals with were underweight	22% of individuals with SCD were stunted		
Sap Ngo Um et al., 2019 [54]	Yaoundé, Cameroon	2–5 years	77	Compared with WHO growth standards	4% of subjects were underweight and 5% of subjects were wasted	4% of subjects were stunted		Trend towards higher rates of underweight, wasting, and stunting with increasing age
Alexandre-Heymann et al., 2019 [55]	Cameroon, Ivory Coast, Gabon, Mali, Senegal	5–21 years	2583; phenotypes included SS, Sβ^0^, SC, Sβ^+^	287 HbAA or HbAS individuals	See “other assessments”	See “other assessments”	See “other assessments”	• The primary outcome of “growth failure” was defined as a height and/or weight and/or BMI below the 5th percentile on WHO 2007 growth charts. • Signifcantly higher rates of growth failure found in individuals with SS and Sβ^0^ phenotypes • Growth failure not found to be correlated with history of SCD-related medical complications • Differences in growth failure rates most pronounced in males aged 15–17 years
Arigliani et al., 2019 [56]	Kaduna, Nigeria	6–18 years	154	364 age-matched controls	Significantly increased rate of wasting in individuals with SCD	Significantly increased rate of stunting in individuals with SCD		
Arigliani et al., 2019 [57]	Kinshasa, Democratic Republic of Congo	6–18 years	112	377 schoolchildren controls	Significantly increased rate of wasting in individuals with SCD	Significantly increased rate of stunting in individuals with SCD		
Ukoha et al., 2020 [58]	Enugu, Nigeria	1–18 years	175	175 age-, gender-, and socioeconomic status-matched HbAA individuals	Significantly lower Z-score for weight-for-age in individuals with SCD, and significantly higher rate of wasting in individuals with SCD (using WHO growth references)	Significantly lower Z-score for height-for-age in individuals with SCD, and significantly higher rate of stunting in individuals with SCD (using WHO growth references)	Significantly lower Z-score for BMI-for-age in individuals with SCD

**Table 2:** Studies of macronutrient or micronutrient levels in individuals living with sickle cell disease in Africa

Authors and year of publication	Location	Ages	No. of subjects	Control group	Nutrient type	Findings
VanderJagt et al., 1997 [22]	Jos, Nigeria	10 months – 14 years (mean 7 years for males; mean 6 years for females)	13	17 age- and gender-matched controls	Proteins/amino acids	• No significant differences in concentrations of total protein, albumin, serum creatinine, or albumin/globulin ratios • Significantly reduced serum prealbumin levels in individuals with SCD • Significantly reduced serum concentrations of all essential amino acids and most non-essential amino acids (exceptions: alanine, glutamic acid, proline**)** in individuals with SCD
Cox et al., 2011 [59]	Dar-es-Salaam, Tanzania	Mean 17–18 years	11 patients who had succumbed	12 age- and gender-matched controls (all patients had SCD; comparison was between those alive and those who had succumbed)	Proteins/amino acids	• Significantly lower BMI, a trend for lower taurine levels, and significantly lower l arginine bioavailability in individuals with SCD who later succumbed • No differences in hemolytic markers (unconjugated bilirubin, lactate dehydrogenase, aspartate transaminase, alkaline phosphate), with the exception that conjugated bilirubin at enrollment was significantly higher in patients who later succumbed compared to those who did not
Enomoto et al., 1998 [60]	Jos, Nigeria	Females mean 6.3 years; males mean 6.8 years	13	14 age-matched controls	Fatty acids	• No difference in proportions of linoleic and α-linolenic fatty acids • Significantly increased levels of palmitic acid and oleic acid in individuals with SCD • Significantly reduced levels of arachidonic acid, eicosapentanoic acid, and decosahexanolc acid
Glew et al., 2002 [61]	Jos, Nigeria	5–17 years (mean 13 years)	77	73 age- and gender-matched controls	Fatty acids	• No differences in levels of linoleic acid • Significantly reduced α-linolenic acid and arachidonic acid in females with SCD; no difference in males • Significantly reduced eicosapentanoic acid and docosahexaenoic acid in individuals with SCD • Significantly increased proportions of palmitic acid (16:0) and oleic acid (18:1n-9) in serum phospholipids in individuals with SCD
VanderJagt et al., 2002 [26]	Jos, Nigeria	Females mean 13.2 years; males mean 13.4 years	72	68 age- and gender-matched controls	Fatty acids	• No differences in linoleic and α-linolenic acid • Significantly reduced long chain polyunsaturated fatty acids and arachidonic acid in individuals with SCD • Significantly higher palmitic acid and oleic acid in individuals with SCD
Glew et al., 2003 [29]	Jos, Nigeria	9–20 years (mean 14 years for males; mean 13 years for females)	77	75 age- and gender-matched healthy controls	Fatty acids	• Significantly reduced linoleic acid, arachidonic acid, α-linolenic acid, eicosapentanoic acid, and docosahexaenoic acid in serum cholesterol esters in individuals with SCD • Significantly increased palmitic acid and oleic acid in serum cholesterol esters in individuals with SCD
Hamdy et al., 2015 [62]	Cairo, Egypt	6–18 years (mean 12 years)	30	30 age- and gender-matched controls	Fatty acids and vitamins	• Significantly reduced cholesterol, triglycerides, and LDL in individuals with SCD • No differences in HDL • Significantly reduced levels of selenium and vitamin E in individuals with SCD
Ren et al., 2008 [63]	Enugu, Nigeria	11–43 years	26	30 HbAA individuals aged 22–53 years	Fatty acids and vitamins	• Significantly reduced eicosapentanoic acid and docosahexaenoic acid in red blood cell choline phosphoglycerides in individuals with SCD • Significantly reduced plasma retinol, α-tocopherol, and β-carotene concentrations, and reduced activity of red cell copper/zinc-superoxide dismutase, in individuals with SCD
Shukla et al., 1999 [64]	Malawi	2–19 years (mean 9 years)	28	No control group; comparisons with normal range (< 8.0 μmol/L)	Vitamins	• Reduced vitamin E levels in 12 children (63%) • Reduced vitamin E/cholesterol ratio in 10 children (36%), indicating vitamin E deficiency
Jiya et al., 2005 [65]	Sokoto, Nigeria	9 months – 12 years (mean 6 years)	27 with HbSS and 11 with HbSS and persistent fetal hemoglobin	32 age- and gender-matched controls	Vitamins	• Significantly lower vitamin A (retinol), vitamin C (ascorbic acid) and vitamin E (α-tocopherol) in individuals with SCD
Cox et al., 2011 [66]	Tanzania	2–15 years (median 8 years)	23	18 siblings aged 2–12 years (median 7 years)	Vitamins	Vitamin C deficiency identified in 48% of individuals with SCD
Tsang et al., 2014 [40]	Nyanza Province, Western Kenya	6–35 months	14	288 individuals from a random sample of 882	Vitamins	• No significant association with vitamin A deficiency
Adegoke et al., 2017 [67]	Ile-Ife, Nigeria	Mean age 7 years	95	75 age- and gender-matched HbAA individuals	Vitamins	Significantly reduced mean serum 25-hydroxy vitamin D in individuals with SCD
Adegoke et al., 2017 [48]	Ilesa, Nigeria	4–11 years (mean 7 years)	95	109 Brazilian children with SCD aged 4–11 years (study compares SCD populations in Nigeria and Brazil)	Vitamins	• Suboptimal vitamin D levels in 12.6% of Nigerian individuals with SCD; none had severe vitamin D deficiency
Adegoke et al., 2017 [68]	Nigeria	1–15 years (mean 8 years)	123	Study examined effect of vitamin D levels on pain (no control group)	Vitamins	• Deficient or insufficient serum 25-hydroxyvitamin D (vitamin D) in 11% of individuals with SCD; none had severe vitamin D deficiency
Siegert et al., 2018 [69]	Uganda	1–4 years	99 individuals with SCD randomly selected from the NOHARM study [70]	Compared with standard reference values	Vitamins	• 53% of children were vitamin D-insufficient (unrelated to inflammation) • Prevalence of vitamin deficiency: vitamin A (18%), vitamin B12 (3%), vitamin D (6%), vitamin E (1%)
Ajayi et al., 1997 [71]	Lagos, Nigeria	Mean 21 years	30 (females only; 10 HbSS, 10 HbAS, 10 HbAC)	10 HbAA individuals	Minerals	• Significantly reduced zinc levels in individuals with SCD compared to heterozygotes and HbAA controls • Significantly reduced mean serum and erythrocyte copper in individuals with SCD compared to heterozygotes and HbAA controls • Significantly reduced serum, erythrocyte, and urine magnesium in individuals with SCD compared to heterozygotes and HbAA controls
Akenami et al., 1999 [72]	Ibadan, Nigeria	16–42 years	35 (23 HbSS, 12 HbSC)	25 age- and gender-matched HbAA individuals	Minerals	• Significantly reduced serum zinc in individuals with HbSS and HbSC • Significantly increased serum copper and magnesium in individuals with HbSS; no difference in individuals with HbSC
Oladipo et al., 2005 [73]	Lagos, Nigeria	7–170 months	86	45 age- and gender-matched HbAA individuals	Minerals	• Significantly increased serum phosphorus in individuals with SCD • Significantly reduced serum calcium in individuals with SCD • No differences in serum magnesium and albumin
Ojo et al., 2006 [74]	Ile-Ife, Nigeria	10–60 years	84 (divided by multiple methods of analysis and sample sites)	141 (divided by multiple methods of analysis and sample sites)	Minerals	• Elevated erythrocyte sodium in individuals with SCD • Significantly reduced potassium, zinc, iron, and riboflavin in whole blood and/or erythrocytes in individuals with SCD
Arinola et al., 2008 [75]	Ibadan, Nigeria	Not stated	20 individuals with HbSS without malaria; 24 individuals with HbSS with malaria	18 HbAA individuals with malaria; 32 HbAA individuals without malaria	Minerals	• Significantly reduced iron, zinc, and magnesium in individuals with SCD compared to controls • Significantly increased urea in non-malaria infected individuals with SCD compared with non-malaria infected controls • Significantly reduced levels of total antioxidants in non-malaria infected individuals with SCD compared with non-malaria infected controls • No differences in magnesium, copper, chromium, cadmium, and selenium in non-malaria infected individuals with and without SCD • No differences in levels of serum albumin
Olaniyi et al., 2010 [76]	Ibadan, Nigeria	26–55 years	59	35 age- and gender-matched controls	Minerals	• Significantly increased mean plasma levels of zinc and nitric oxide in individuals with SCD • Significantly reduced levels of serum iron, chromium, and selenium in individuals with SCD • No differences in levels of magnesium, manganese, and copper
Cox et al., 2012 [77]	Tanzania	3–15 years (mean 8 years)	32	No control group	Minerals	Nocturnal hemoglobin oxygen desaturation in individuals with SCD associated with higher transferrin saturation
Onukwuli et al., 2017 [52]	Enugu, Nigeria	6–18 years	81 (females only)	81 age- and socioeconomic class-matched HbAA individuals from outpatient clinic	Minerals	Significantly reduced levels of serum zinc in individuals with SCD
Sungu et al., 2018 [78]	Kasumbalesa, Democratic Republic of Congo	2–15 years (mean 10 years)	76	76 age-, gender-, and residence area-matched controls	Minerals	Significantly reduced levels of zinc and magnesium in individuals with SCD
Lee et al., 2018 [79]	Tanzania	3–18 years	199	No control group	Minerals	Lower hepcidin in more severely anemic individuals with SCD
Ajibola et al., 2019 [80]	Osun State, Nigeria	Median age 24 years	60 individuals with phenotypes SS or SC	83 HbAS or HbAC individuals; 50 HbAA individuals	Minerals	• Malondialdehyde and superoxide dismutase significantly higher in Hb variants compared to controls • Glutathione and total antioxidant stats levels significantly reduced in Hb variants • Overall results suggested that SCD patients & carriers were more vulnerable to oxidative stress
Emokpae et al., 2019 [81]	Benin City, Nigeria	4–20 years	100 HbSS individuals	50 age- and gender matched HbAA individuals	Minerals	Significantly higher serum copper levels and significantly lower zinc levels in individuals with SCD compared to controls
Antwi-Boasiako et al., 2019 [82]	Accra, Ghana	Mean ages ranged 21–38 years old (depending on phenotype)	90 HbSS and HbSC individuals	50 HbAA individuals	Minerals	• Significantly higher serum iron and copper in individuals with SCD compared to controls • Serum iron and copper were further increased in patients with HbSS and vaso-occlusive crises • Serum zinc levels were significantly lower in individuals with SCD, especially during vaso-occlusion
Kudirat et al., 2019 [83]	Kano, Nigeria	6 months-15 years	140 (70 with acute pain crises, 70 in steady state)	70 HbAA individuals	Minerals	Significantly lower serum zinc level in individuals with SCD compared to controls, which was made worse during vaso-occlusive crises
Erhabor et al., 2019 [84]	Sokoto, Nigeria	1–15 years	45	25 age-matched HbAA individuals	Minerals	Significantly lower mean serum copper and selenium in individuals with SCD

**Table 3:** Studies of nutritional interventions involving individuals living with sickle cell disease in Africa

Authors and year of publication	Location	Ages	No. of subjects	Intervention	Nutrient intervention type	Design	Outcome	Comments
Afolabi et al., 2012 [85]	Lagos State and Ogun State, Nigeria	15–48 years	Not reported	Seed oils from *Solenostemon monostachyus, Ipomoea involucrate* and *Carica papaya* plants	Plant extract/ seed oil	In vitro comparisons using blood from SCD patients; comparison groups were controls, cells treated with glutathione, and cells treated with a known anti-sickling plant extract	• All plant extracts studied led to reduction in sickled red blood cells, reduction in Fe^+ 2^/Fe^+ 3^ ratios, and reduction in lactate dehydrogenase activity when compared with controls • Individual extracts also showed varying improvements in hemoglobin concentration, sickle cell polymerization inhibition, and catalase activity	Some gender dependent differences were noted; specific bioactive compounds within each plant extract were not isolated
Imaga et al., 2013 [86]	Lagos State, Nigeria	15–28 years	4 (2 treated, 2 controls)	Oral ingestion for two weeks of a commercial product made from *Cajanus cajan* plant extract	Plant extract/ seed oil	Non-blinded in vitro comparison study	• Treatment group reported to have anti-sickling effect, but no statistical analyses performed • Treatment group reported to have increased fetal hemoglobin, but no statistical analyses performed • No effect reported on packed cell volume, hemoglobin, or mean corpuscular hemoglobin	Statistical analyses lacking for main outcomes
Kaddam et al., 2019 [87]	Khartoum, Sudan	5–42 years	47	*Acacia Senegal* (gum Arabic) supplementation as a lipid-lowering agent	Plant extract/ seed oil	Single-arm trial	Treatment led to significantly decreased total cholesterol, triglycerides, and low-density lipoprotein; no effect on high-density lipoprotein	Gum Arabic is a dried, gummy substance obtained from the acacia Senegal tree
Adegoke et al., 2013 [88]	Ekiti State, Nigeria	1 year to “adolescent” (upper range not specified)	125 (65 treated, 60 controls)	Oral ingestion for 6 months of freshly squeezed lime juice; dose was weight- dependent (range: 10–30 ml daily)	Micronutrient	Open label, randomized study	• Treated group had significantly fewer pain episodes, febrile illness, and admission rate • Treated group had significantly increased mean hematocrit • No change in transfusion rate, organomegaly, or jaundice	Effect hypothesized to result from vitamin C, amino acids (especially phenylalanine) and flavonoids
Adegoke et al., 2017 [67]	Ile-Ife, Nigeria	Mean age 7 years	170 (95 treated, 75 controls)	Oral ingestion for 3 months of vitamin D supplementation in children with SCD that had low 25-hydroxyvitamin D (25-OHD) levels	Micronutrient	Age- and gender-matched controlled study	• Children with SCD and low 25-OHD levels had enhanced levels of pro-inflammatory cytokines • Treatment with vitamin D in children with SCD that had low 25-OHD levels led to an improved pro-inflammatory cytokine profile	Effect hypothesized to result from anti-inflammatory influence of supplemental vitamin D
Daak et al., 2013 [89]	Khartou, Sudan	2–24 years	128 (67 treated, 61 controls)	Oral ingestion for 1 year of Omega-3 capsules containing EPA and DHA fatty acids; dose was weight-dependent	Macronutrient	Double-blinded, placebo-controlled, randomized study	• Treatment group had improved primary outcome: significantly fewer clinical vaso-occlusive events • Treatment group also had reduced severe anemia, reduced blood transfusions, reduced white blood cell counts, and reduced school absences due to disease • No change in rates of stroke, sequestration crisis, or vascular necrosis	The study was not powered to detect changes in rates of stroke, sequestration crisis, or vascular necrosis
Cox et al., 2018 [90]	Dar-es-Salaam, Tanzania	8–12 years (mean 10 years)	119	Oral ingestion of ready-to-use supplementary food (RUSF) with and without arginine and citrulline	Macronutrient	Double-blind, random order crossover trial	• RUSF increased the global arginine bioavailability ratio and improved measures of endothelial function, and led to improvements in growth • RUSF fortified with arginine and citrulline did not additionally increase the plasma global arginine bioavailability ratio or improve endothelial function	Arginine is the sole substrate of endothelial nitric oxide synthase and has been implicated in pathophysiology of SCD complications
Onalo et al., 2019 [91]	Abu, Nigeria	5–17 years (mean 11 years)	68 (35 treated, 33 controls)	Oral arginine therapy every 8 h until discharge in SCD patients hospitalized with severe vaso-occlusive events	Macronutrient	Double-blind, randomized, phenotype included placebo-controlled trial	• Plasma arginine levels increased by 125% (arginine arm) vs 29% (placebo arm) • 54% of children treated with arginine were discharged compared to 24% in placebo arm by day 5 • Arginine treatment appeared to ameliorate some measures of pain • No significant differences in adverse events but arginine arm trended more towards vomiting compared to placebo	Arginine had previously been shown to have benefits in individuals with SCD in studies performed in the Unite States

### Descriptive studies of anthropometric characteristics

Nearly one-half of all studies identified focused on anthropometric characteristics. The studies typically assessed height, weight, and body mass index (BMI). Other measurements included head circumference, arm span, and various body composition parameters.

The majority (25/37; 68%) of studies were conducted in Nigeria. Other studies involved populations in Democratic Republic of Congo (DRC) [49, 50, 57], Ghana [33, 53], Tanzania [32], Egypt [23], Cameroon [54], and Algeria [37]. In addition, two multi-country studies involved patients in Cameroon, Ivory Coast, Gabon, Mali, and Senegal [43, 55]. The majority of reports focused on children and adolescents; only one study exclusively involved adults [24]. Most studies evaluated approximately 50–200 patients and a similar number of age- and gender-matched healthy controls; three large studies enrolled over 1000 SCD patients each [32, 43, 55]. Several studies used WHO growth standards for comparison rather than a non-sickle cell disease control group. Male and female subjects were generally equally represented in the study populations.

The three largest studies found significant growth defects compared to healthy controls. A multi-country study of more than 3500 SCD patients (aged 10–24 years) with nearly 1000 controls in Cameroon, Ivory Coast, Gabon, Mali, and Senegal was designed to evaluate determinants of vascular complications [43]. Anthropometric analyses showed that SCD patients were significantly shorter and had lower BMI than controls; weight was not reported. A caveat of the study was that the control group was significantly older (median age 24 vs 16 for patients) and more likely to be female (60% vs 54% for patients) than the SCD group. A follow-up study involving the same subject population revealed significantly higher rates of growth failure, defined as a height and/or weight and/or BMI below the 5th percentile using WHO growth reference [55]. Another large study followed a cohort of approximately 1000 SCD patients aged 6 months to 48 years over 5 years and found SCD to be significantly associated with stunting, underweight, and wasting, with the most pronounced effects associated with adolescent age and male gender [32]. Adult men were seven times more likely than adult women to be underweight and were significantly more likely to be stunted and wasted. Females demonstrated improved catch-up growth compared with males following growth deficits that were identified during adolescence.

The studies involving smaller sample sizes showed greater variation in the results, but some trends emerged. Several reports confirmed the finding that males were more likely to show growth defects than females [22, 27, 29, 45]. Multiple studies also noted that growth deficits became more pronounced with age. For example, a study that involved young patients aged 6–35 months showed no association with wasting, stunting, or underweight status [40]. A study of 233 children aged 2–17 years with SCD in Lagos, Nigeria found that the factor most significantly associated with both wasting and stunting was older age [45]. Additional studies similarly detected one or more growth deficits in adolescents but not in younger children [25, 28, 34, 39, 41, 46]. In several Nigerian studies, SCD patients were found to be underweight or to have low BMI, but showed no difference in height compared to controls [22, 25, 35, 42, 44, 52]; while other Nigerian studies showed differences in both weight and height [24, 26, 27, 29, 44, 51]. These variable findings may have resulted from the fact that the studies were not powered to detect significant differences in height specifically. Reports from DRC, Egypt, and Ghana found that children with SCD had a higher prevalence of stunting compared to controls, but did not always show differences in wasting or BMI [23, 33, 50]. SCD was also associated with delayed puberty [23, 31]. Three studies from Nigeria reported the presence of overweight and obesity among patients with SCD although in lower proportions (an average of less than 3% of the sample population) [36, 42, 45].

### Descriptive studies of macro- or micronutrient status

The second most common group of studies identified involved assessment of biomarkers from serum samples for macronutrients or micronutrients. Most studies were conducted in Nigeria (21/31, 68%); other studies took place in DRC [78], Tanzania [32, 77, 79], Egypt [62], Kenya [40], Ghana [82], Uganda [69], and Malawi [64]. Nutritional parameters measured included proteins/amino acids, fatty acids, vitamins, and minerals. Most studies included less than 100 individuals with SCD. Both children and adults were studied, with male and female subjects generally equally represented.

Serum protein levels were investigated in one small study (13 children with SCD and 17 healthy controls) in Nigeria in which no significant differences were reported in the concentrations of total protein or albumin between SCD patients and controls [22]. However, serum prealbumin levels were significantly lower for the population of patients with SCD, which was hypothesized to result from poor nutrition or existing disease-related inflammation. The serum concentrations of all amino acids except alanine, glutamic acid, and proline were significantly reduced in SCD patients. A small study involving 23 participants in Tanzania measured the steady state nutrition status of SCD patients who later died (*n* = 11) compared with those who were alive at the end of the study period. Those who suffered mortality had a significantly lower BMI, plasma taurine levels and arginine bioavailability before succumbing [59].

Proportions of fatty acid and the state of metabolism were evaluated in four related studies of young SCD patients in Nigeria [26, 29, 60, 61]. These reports found perturbed pathways of fatty acid elongation and desaturation in children with SCD. Specifically, arachidonic acid, eicosapentanoic acid (EPA), and decosahexanoic acid (DHA) were significantly reduced, whereas saturated (palmitic acid) and monounsaturated (oleic acid) were significantly elevated in patients compared to controls. Another study in a population of 26 SCD patients aged 11–43 in Enugu, Nigeria, confirmed the finding that EPA and DHA fatty acids are reduced in SCD patients [63]. The authors of these studies hypothesized that reduced polyunsaturated fatty acids in the phospholipids of the cell membrane of SCD patients could lead to their being more rigid, thereby contributing to disease symptoms. A study of 30 children with SCD in Egypt found that patients also had significantly lower cholesterol, triglycerides, and LDL (but not HDL) in blood plasma compared to healthy controls [62].

Acknowledging that interpretation of plasma concentrations of vitamins and minerals can be problematic in patients with ongoing inflammation, analysis of serum vitamin levels in SCD patients generally indicated lower concentrations of vitamin A [63, 65] vitamin C [65, 66] and vitamin E [62–65]. One study of 14 SCD patients in Kenya found no association of HbSS phenotype and low concentrations vitamin A [40]. Three reports of a related study population in Ilese, Nigeria, investigated vitamin D status of young SCD patients [48, 67, 68]. When compared to healthy controls, mean 25-hydroxyvitamin D levels were significantly lower in SCD patients and suboptimal vitamin D levels were seen in greater than 10% of patients. However, no SCD patients with severe vitamin D deficiency (defined as < 20 ng/ml) were observed. A limitation of the latter two studies was the lack of a healthy comparator group; each used vitamin D deficiency cut-off values for a healthy population in other published studies as reference.

Selected minerals were evaluated in eight small studies of SCD patients and compared to healthy controls. Serum iron concentration was reduced in patients compared to controls in all studies that evaluated it [74–76, 82]. Serum or plasma zinc was also generally reduced in SCD patients [7, 71, 72, 74, 75, 78, 83], although zinc was elevated in one population of 59 Nigerian adult SCD patients [76]. Measures of other minerals showed mixed results. Magnesium levels were either reduced [71, 75], elevated [72] or unchanged [73, 75, 76] in SCD patients compared to healthy controls. Similarly, copper was reduced [71], elevated [72, 81, 82] or unchanged [75, 76] in SCD patients. Other minerals measured in only a few studies included manganese, chromium, selenium, potassium, rubidium, cadmium, and calcium.

### Interventional studies

A very small number of clinical studies involving nutritional interventions in SCD patients in African countries were identified. There were four randomized trials [88–91]. The first was a study of 125 SCD patients in Nigeria (involving children aged 1 year and above) that tested the effect of lime juice on SCD parameters. All patients in the treatment group (*n* = 65) as well as controls (*n* = 60) were given folic acid, vitamin B complex, and proguanil, with the treatment group also given twice-daily oral lime juice with weight-based dosing ranging from 5 to 15 mL. Each child was assessed monthly for 6 months. The group receiving lime juice was reported to have significantly fewer pain episodes, febrile illnesses, and hospital admission rates. There was no change in transfusion rate, organomegaly, or jaundice. The positive effect was postulated to result from vitamin C, amino acids (in particular, phenylalanine), and flavonoids contained in the juice, but no direct evidence for this was provided [88].

A second study of 128 SCD patients aged 2–24 years in Sudan investigated the impact of 1 year of treatment with oral omega-3 capsules containing EPA and DHA fatty acids (using age- and weight-dependent dosing) compared to placebo**.** The hypothesis was that omega-3 fatty acids could reduce red blood cell aggregation, adherence, and inflammation that occur during sickle cell disease-mediated vaso-occlusive crises. The treatment group had significantly fewer clinical vaso-occlusive events, as well as reduced rates of severe anemia and need for blood transfusions. The study was not powered to detect changes in other outcomes such as stroke, sequestration crisis, or vascular necrosis [89].

A third trial utilized a double-blind, random order design. Ready-to-use-supplementary food was studied in 119 children with SCD in Tanzania [90]. Two different formulations of the supplements were compared: a commercially available (Nutriset, France) ready-to-use-supplementary food (RUSF) fortified with vitamins and minerals according to recommended daily allowances and an “enhanced” version of the same RUSF (providing 500 kcal/day) that was additionally fortified with arginine and citrulline. Arginine is the substrate for endothelial nitric oxide synthase, a natural vasodilator, and has been implicated in pathophysiology of SCD complications. In the cross-over study design, children received each treatment for 4 months, with 4-month washout periods following the intervention. Ready-to-use-supplementary food led to small weight gains, an increased arginine bioavailability ratio, and improved measures of endothelial function compared to baseline; addition of arginine and citrulline to the supplement did not provide additional benefits [90].

The final randomized study identified involved the regular administration of oral arginine therapy to 35 hospitalized patients with SCD in Nigeria and compared the effects with 33 control subjects. Plasma arginine levels increased by 125% in the arginine arm compared with 29% in the control arm [91]. Arginine treatment was associated with quicker discharge and reduced pain events. The rate of adverse events was non-significant between the two treatment arms, however there was a trend towards increased vomiting in the patients treated with arginine. A previous study outside of Africa also found positive clinical effects associated with the use of arginine [92].

One of the non-randomized interventional studies identified investigated the use of vitamin D supplementation. A small treatment arm was nested in a Nigerian study comparing blood levels of vitamin D and pro-inflammatory cytokines [48]. The hypothesis was that low vitamin D levels might lead to a pro-inflammatory environment that exacerbates SCD symptoms. Twelve children with SCD who were determined to have low vitamin D levels were given 3 months of oral vitamin D supplementation (2000 U). At the end of treatment, mean serum 25-hydroxyvitamin D levels were significantly increased compared to baseline, levels of several proinflammatory cytokines were significantly decreased, and the levels of anti-inflammatory cytokine IL-11 were significantly increased.

## Discussion

To our knowledge this is the first review of nutrition-related studies involving individuals living with SCD in sub-Saharan Africa. While a moderate number of studies were identified, most were descriptive in nature and small in terms of numbers of subjects. Approximately two-thirds of studies took place in a single country (Nigeria). In addition, there were very few interventional trials designed to measure the impact of an isolated nutritional intervention and only four randomized studies. The findings of this review suggest an outstanding need for nutrition-focused research relating to the care of individuals with SCD in Africa, with a particular emphasis on research with practical implications for clinical management in order to improve patient outcomes.

The findings of studies identified through this review are generally consistent with nutrition-related investigations involving SCD patients in other parts of the world. More than 50 years ago, poor growth was first reported in patients with SCD, and that observation has since been repeated in multiple studies involving SCD populations in Jamaica, Brazil, India, and North America [13, 15, 93–99]. Many of these studies specifically note that the growth faltering occurred in patients that were receiving recommended daily protein and calorie intakes. The pathophysiology of growth problems in SCD patients has come into sharper focus in recent decades. A leading view is that the increased rate of red cell turnover, a primary feature of SCD patients, underlies a hypermetabolic state. The biochemical and physiological factors that contribute to hypermetabolism include increased protein turnover, increased myocardial activity, and production of proinflammatory cytokines [100–104]. The supposition is that the energy and nutrient requirements normally recommended are not adequate in patients with SCD given their increased energy expenditures and other unusual metabolic demands, which compete directly with energy needs required to sustain adequate growth.

Evidence derived from robust interventional studies is important to support recommendations for specific nutrition-related practices for patients with sickle cell disease. Only four randomized trials were identified. The studies were small, each involving less than 150 individuals with SCD. Positive clinical benefits were found with the use of lime juice, long-chained fatty acid supplementation, RUSF, and oral arginine; ideally these findings would be confirmed in larger follow-up investigations. It is worth noting the paucity of robust interventional trials designed to test the effect of macronutrient supplementation in individuals with SCD despite the evidence, as described above, that nutrition deficits in this population are likely to be caused at least in part from the increased energy demands that result from altered metabolism.

Guidelines for clinical management of patients with SCD published by internationally recognized organizations do not provide special guidance for nutritional care [105, 106]. Given that the risk of poor growth in SCD patients is increasingly reported, and the fact that there is plausible pathophysiologic drivers of nutritional disturbances in SCD patients, there appears to be a substantial gap in research in this area to inform much needed evidence-based recommendations.

Limitations of this systematic review include the fact that nearly half of studies identified were largely anthropometry-based descriptive studies. Few studies involving nutrition interventions in sub-Saharan Africa were identified, only several had robust methodologies, and none have been validated in repeated studies. In addition, the studies involving analyses of vitamin and mineral levels in SCD patients in sub-Saharan Africa overall involved small numbers of patients and generally were unable to link findings with meaningful clinical correlations in ways that might influence nutritional care practices. Another limitation is that most investigations identified took place in the single country of Nigeria (at the same time, acknowledging that Nigeria is home to the largest population of SCD patients globally).

## Conclusion

Despite the reality that most SCD patients globally live in sub-Saharan Africa, and the fact that nutritional disturbances in SCD patients are increasingly well described, there has been limited research focused on ways that nutritional care might help to improve clinical outcomes in this patient population. A systematic review of the literature revealed studies that consistently reported stunted growth and malnutrition in African SCD patients during childhood and adolescence, but failed to identify robust, validated studies that could be used to inform clinical management. Our study suggests an outstanding need to determine if and how supportive nutritional care can reduce disease severity and improve health outcomes for individuals with SCD in sub-Saharan Africa. As such, priority research in this area in the future may include systematic assessment of the drivers of nutritional status in SCD patients, studies that directly advance the understanding of macro- and micronutrient deficiencies associated with clinically significant physiologic effects, and investigations that evaluate the impact of nutritional interventions to inform evidence-based nutrition guidance.

## Data Availability

All data generated or analyzed during this study are included in this published article.

## Abbreviations

SCD: Sickle cell disease
EPA: Eicosapentanoic acid
DHA: Decosahexanoic acid
PRISMA: Preferred reporting items for systematic reviews and meta-analysis
